# Supplementary material for: Preclinical Assessment of Dactinomycin in KMT2A-Rearranged Infant Acute Lymphoblastic Leukemia
Source: Cancers (Basel). 2025 Feb 5;17(3):527. doi: 10.3390/cancers17030527 (PMC11816686; doi:10.3390/cancers17030527)
Supplement: Supplementary file 1 [file cancers-17-00527-s001.zip › cancers-3408626-supplementary.pdf]

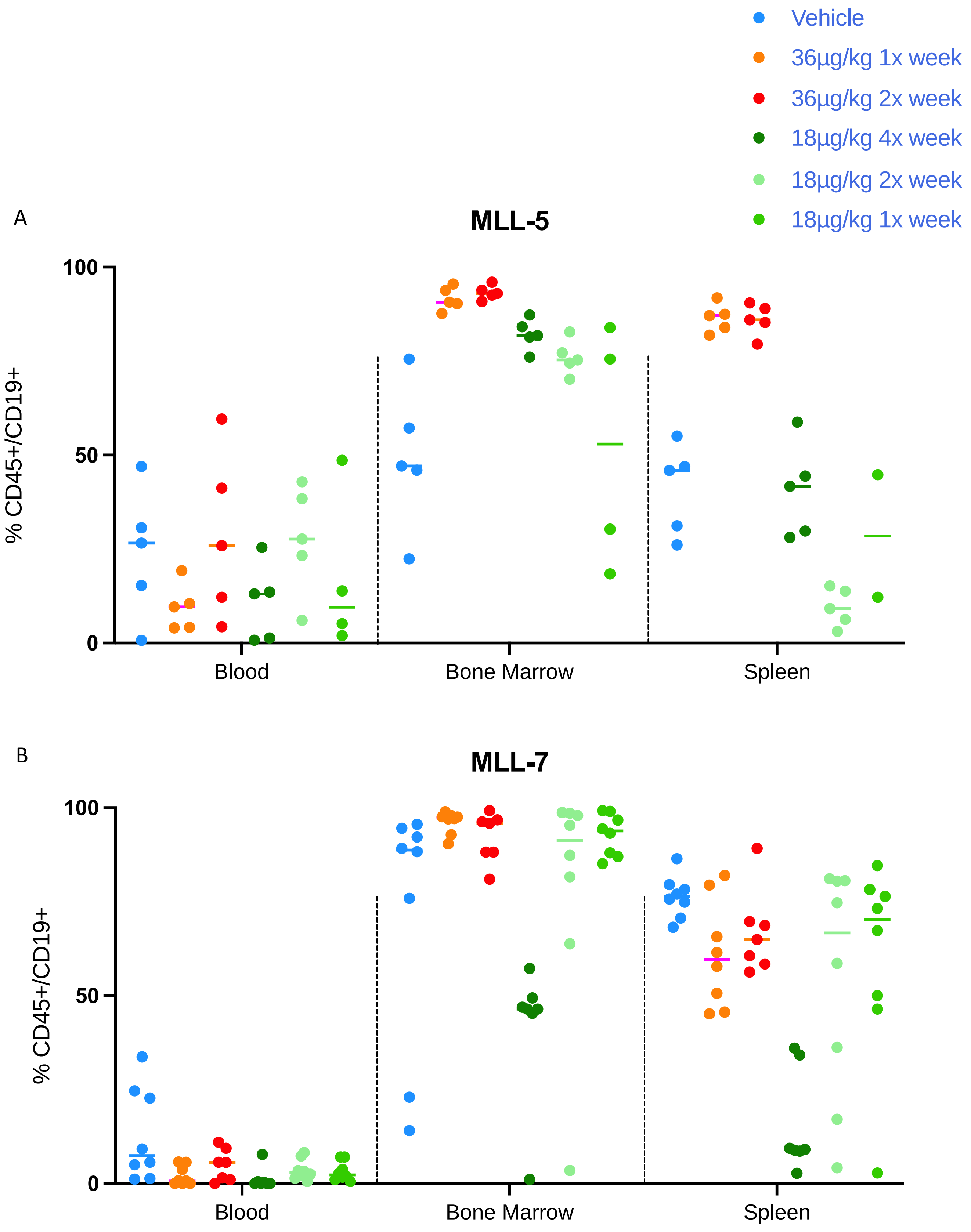

Supplemental Figure S1. Leukemia percentage at endpoint of mice transplanted with (A) MLL-5 and (B) MLL-7 treated with dactinomycin or vehicle control by flow cytometry. Leukemia percentage was calculated by numbers of human CD45/CD19 positive cells relative to total viable mononuclear cells.
